# Supplementary material for: Patterns of Intron Gain and Loss in Fungi
Source: PLoS Biol. 2004 Nov 30;2(12):e422. doi: 10.1371/journal.pbio.0020422 (PMC532390; doi:10.1371/journal.pbio.0020422)
Supplement: Table S1 — Also available at http://genes.mit.edu/NielsenEtAl/. (4.3 MB ZIP). [file pbio.0020422.st001.zip › NielsenEtAl/html/353.html]

AN0720.1.NCU07266.1.MG10496.1.FG09628.1


```
 CLUSTAL W (1.82) Multiple Sequence Alignments - Introns Inserted


Sequence 1: NCU07266.1	287 aa
Sequence 2: MG10496.1	293 aa
Sequence 3: FG09628.1	287 aa
Sequence 4: AN0720.1	304 aa
Alignment Length: 313 aa
Number Identitical Residues: 173 aa
Alignment Score (without introns) 7988


MG10496.1 	MLYLIGLGLSDETDITVKGLEIVKTAARVYLEAYTSILLVDQKVL0ESYYGRSIEVADRE
NCU07266.1	MLYLVGLGLSDETDITVKGLEVVKKAERVYLEAYTSILLVDQATL0ESYYGRPIVVADRE
FG09628.1 	MLYLVGLGLSDETDITVKGLEVVKKASRVYLEAYTSILLVEQSVL0ESYYGRSITVADRE
AN0720.1  	MLYLVGLGLADERDITVRGLEVVKKAERVYLEAYTAILLVDKAKL0EAFYGRPVIEADRE
          	****:****:** ****:***:**.* ********:****::  * *::***.:  ****

MG10496.1 	MVESNSDEILRNAATEDVAFLVVGDPFG2-------------------ATTHTDLVIRAR
NCU07266.1	MVESNSDEILRDADKVDVAFCVVGDPFG~-------------------ATTHTDLVLRAR
FG09628.1 	MVESNSEEILRNAQNEDVAFLVVGDPFG2-------------------ATTHTDLVLRAR
AN0720.1  	LVETGSDDILANADKVDVAFLVVGDPFG~YAFYILDMVFDINSIRAFRATTHTDLVLRAR
          	:**:.*::** :* . **** *******  :    .   . .:  :  ********:***

MG10496.1 	QLQIPIRTIPNASIMSAIGACGLQLYNFGQTVSMVFFTENWRPSSFYDRVAENRALGLHT
NCU07266.1	ELGIQVRTVPNASIMSGIGAAGLQLYNFGQTVSMVFFLDNWRPASFYDRIKENRSIGLHT
FG09628.1 	ELEIPVRTVPNASIMSGIGACGLQLYNFGQTVSMVFFTDTWKPASFYDRIKENRQIGLHT
AN0720.1  	ELGIESKVIPNASIMSGIGCTGLQLYNFGQTVSMVFFTETWKPSSYYDRVKENVQIGLHT
          	:* *  :.:*******.**. **************** :.*:*:*:***: **  :****

MG10496.1 	LVLLDIKVKEPNFESLARGKLVYEPPRFMTVGTCARQMLEVEEEKVKDGAEGGVCGGEAL
NCU07266.1	LVLLDIKVKEQSLENMARGRKIYEPPRYMTVGTCAQQMLEIEEEKQE-----GVYGPESL
FG09628.1 	LVLVDIKVKEQSLENMARGRLVYEPPRYMTVGQCAQQMLEIEEERKE-----GVYAKDSL
AN0720.1  	LVLLDIKVKEQSLENMARGRLIYEPPRFMTVAQCAAQMLETEEERQE-----GVWGPDSL
          	***:****** .:*.:***: :*****:***. ** **** ***: :     ** . ::*

MG10496.1 	AIGAARVGGKTEKFVAGTLRELADGADELLGAPLHSLVLLGKKTHELEHDFVREFALNQD
NCU07266.1	AIGCARVGGKTEKFVSGTLKELCD-ADDLLGPPLHSLILLGRRTHELEHDFVREFAVNKE
FG09628.1 	AIGAARVGGRTEKFVAGTLEELCS-TDEELGPPLHSLVLLGRRTHELELDYVRQFAVDKE
AN0720.1  	AVGAARVGAEDQKLVAGTLQELTQ---VDMGRPLHSLVLLGRRAHDLEKDYIRRFAVDEA
          	*:*.****.. :*:*:***.** .     :* *****:***:::*:** *::*.**::: 

MG10496.1 	NWDRIWK-ADYQIQP
NCU07266.1	NWDRIWK-AEYCGKQ
FG09628.1 	KWDKIWN-AEYGKQL
AN0720.1  	TFDAAWQNGKYGSS-
          	.:*  *:...*  .
```
